# Rural–urban disparities in the utilization of mental health inpatient services in China: the role of health insurance

**DOI:** 10.1007/s10754-018-9238-z

**Published:** 2018-03-27

**Authors:** Junfang Xu, Jian Wang, Madeleine King, Ruiyun Liu, Fenghua Yu, Jinshui Xing, Lei Su, Mingshan Lu

**Affiliations:** 1 Research Center for Public Health, School of Medicine, Tsinghua University, Beijing, China; 2 Center for Health Economic Experiments and Public Policy, Department of Social Medicine and Administration, School of Public Health, Shandong University, No. 44 Wen Hua Xi Road, Jinan, Shandong China; 3 School of Public Policy and Management, Tsinghua University, Beijing, China; 4 Shandong Center for Mental Health, Jinan, Shandong China; 5 Shandong Health and Family Planning Commission, Jinan, Shandong China; 6 Department of Economics, University of Calgary, Calgary, Canada

**Keywords:** D12, H55, I18

## Abstract

Reducing rural–urban disparities in health and health care has been a key policy goal for the Chinese government. With mental health becoming an increasingly significant public health issue in China, empirical evidence of disparities in the use of mental health services can guide steps to reduce them. We conducted this study to inform China’s on-going health-care reform through examining how health insurance might reduce rural–urban disparities in the utilization of mental health inpatient services in China. This retrospective study used 10 years (2005–2014) of hospital electronic health records from the Shandong Center for Mental Health and the DaiZhuang Psychiatric Hospital, two major psychiatric hospitals in Shandong Province. Health insurance was measured using types of health insurance and the actual reimbursement ratio (RR). Utilization of mental health inpatient services was measured by hospitalization cost, length of stay (LOS), and frequency of hospitalization. We examined rural–urban disparities in the use of mental health services, as well as the role of health insurance in reducing such disparities. Hospitalization costs, LOS, and frequency of hospitalization were all found to be lower among rural than among urban inpatients. Having health insurance and benefiting from a relatively high RR were found to be significantly associated with a greater utilization of inpatient services, among both urban and rural residents. In addition, an increase in the RR was found to be significantly associated with an increase in the use of mental health services among rural patients. Consistent with the existing literature, our study suggests that increasing insurance schemes’ reimbursement levels could lead to substantial increases in the use of mental health inpatient services among rural patients, and a reduction in rural–urban disparities in service utilization. In order to promote mental health care and reduce rural–urban disparities in its utilization in China, improving rural health insurance coverage (e.g., reducing the coinsurance rate) would be a powerful policy instrument.

## Introduction

Mental health has become an increasingly significant public health concern worldwide. Yet the utilization rates of mental health services remain low compared with those for physical health conditions (Simning et al. 2012). Many psychiatric patients do not receive appropriate treatment services at an early stage, which significantly affects the progression of the disease (WHO 2001). A study in the United States found that a median delay of 10 years after the onset of mental illness and before a patient’s first contact with a general practitioner and 11 years before a first contact with a psychiatrist (Wang et al. 2004). This delay in treatment can lead to increased morbidity and mortality, including the development of various psychiatric and physical comorbidities and the adoption of life-threatening and life-altering self-treatments (e.g., licit and illicit substance abuse) (Wang et al. 2007a, b). There is a broad similarity in the challenges faced by mental health care practitioners across various countries (WHO 2001), primarily because mental health care services are rarely covered by health insurance packages and health plans to the same degree as are physical health care services, especially in low- and middle-income countries (Kellermann 2002; Rowan et al. 2013). Not only are more mental health services excluded from insurance coverage, but the eligible mental health services are often subject to higher co-pays and are capped at a maximum number of covered treatments (Sturm 2000).

**Fig. 1:**
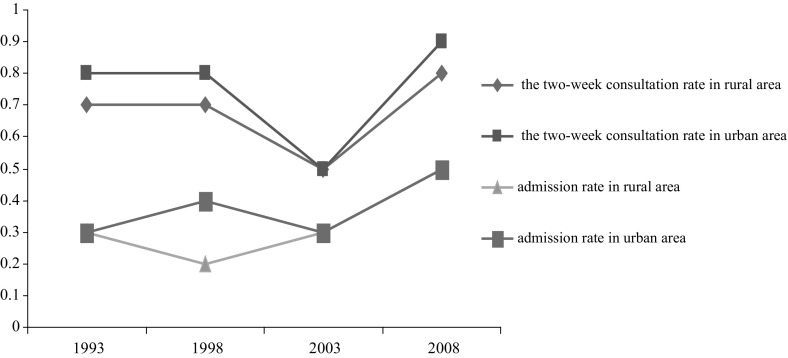
Overall mental health service utilization trend in urban and rural areas in China, 1993–2008 (‰). *Source:* 2013 China Health Statistics Yearbook

In China, for various cultural, socioeconomic, and health-care-related reasons, people with mental health needs have long been under-served (Xu et al. 2014). As shown in Fig. 1, statistics show that the utilization of mental health services remains low in both urban and rural areas. Overall, a two-week consultation rate (the ratio of the visits number among respondents to the total number of respondents within 2 weeks) is about 0.7% and the admission rate ranges from 0.2 to 0.5% (NHFPC 2013). Figure 1 also indicates that the utilization of mental health services in rural areas was even lower than that in urban areas.

Income-related disparities in health care have been a major policy concern in China. In 2001, total health expenditure in China was 502.5 billion RMB (4.6% of gross domestic product, GDP), 60% of which was out-of-pocket (OOP) expenses (CNHEI 2003). Moreover, a health-care reform that started in the 1980s led to the collapse of the Cooperative Medical Scheme (CMS), a collective-economy and prepaid health security system in rural China. Rural health care reverted back to being primarily privately financed. Most rural residents, who at the time accounted for almost 60% of China’s total population, did not have any form of health insurance before 2003 (Li et al. 2014). With most health resources allocated to urban areas and many rural residents not being able to afford high medical costs, rural–urban disparities in health and the utilization of health services increased (Hu et al. 2008). In order to reduce these disparities, the Chinese government implemented the New Cooperative Medical Scheme (NCMS) among the rural population in 2003 (Wagstaff and Van Doorslaer 1992; Wei et al. 2015). As for urban residents, previous public health insurance schemes were integrated into the Urban Employee Basic Medical Insurance (UEBMI) and the Urban Resident Basic Medical Insurance (URBMI) (Liu et al. 2014). By the end of 2012, 89% of urban residents and 97% of rural residents were covered by one of the country’s three main public health insurance schemes (UEBMI, URBMI, and NCMS), up from only 55% of urban residents and 21% of rural residents insured in 2003 (NHFPC 2015).

As of 2009, severe mental illnesses were incorporated into the national public health service program (Xu et al. 2014). In 2012, the Mental Health Law of the People’s Republic of China was passed. Under this law, medical expenses for mental illnesses, just like common illnesses, should be covered by the three basic types of public health insurance (TCPGPRC 2012). However, there are large variations in the actual reimbursement ratios (RRs) for mental health care, partly depending on the type of health insurance. Even for people covered by the same insurance scheme, coverage could still be different based on factors such as age, sector of work, and whether they are retired or not (Yuan et al. 2014). In addition, health insurance in China is not portable; one has to bear the full medical expenses if one is seeking health care outside of one’s province or city of residence. Table 1 details the three main public health insurance policies in China and the different insurance policies available for mental illnesses (deductible amount, annual limit, RRs). As can be seen, the RR is still rather low, particularly for rural areas: 30–70% under NCMS, versus 80–100% under UEBMI (Table 1).

**Table 1:** Rural and urban health insurance schemes, and insurance benefits for inpatient mental care in China

Items	Rural insurance	Urban insurance	
	NCMS	URBMI	UEBMI
Year of launch	2003	2007	1999
Enrollment unit	Household	Household	Individual
Enrollment type	Voluntary	Voluntary	Compulsory
Managed level	County/district	Municipal	Municipal
Managed institution	National heath and family planning commission of China	Ministry of human resources and social security	Ministry of human resources and social security
No. of enrollees	832 million	271.2 million	264.7 million
Financing	410 RMB per person (government contributions: 320 RMB)	*Children and students:* 100 RMB per person (government contributions: 40 RMB); *People over 70:* 560 (government contributions: 440 RMB); *Special population*: all contributions from government; *Other*: 560 (government contributions: 230 RMB)	The employer contributes 5–7% of the employee’ salary while the employee contributes 2%
Accounts	Risk pooling of outpatient services; Risk pooling of inpatient services	Social pooling account (all funds) for inpatient utilization and critical (i.e. chronic or fatal disease) outpatient utilization	Medical Savings Account (including employee contributions and 25–35% of employer contributions) for outpatient utilization; Social Pooling Account (70% of employer, contributions) for inpatient utilization and critical (i.e. chronic or fatal disease) outpatient utilization
Benefit	*Deductible*: 300–2000 RMB *Annual limit*: 50,000–80,000 RMB per year *Reimbursement ratio:* 30–70%, different in different regions.	*Deductible:* 300–900 RMB *Annual limit:* 50,000–160,000 RMB per year *Reimbursement ratio:* 50–70%, different in different regions.	*Deductible:* 400–1200 RMB; *Annual limit:* 55,000–290,000 RMB per year *Reimbursement ratio:* 80–100%, different in different regions
Payment method	Fee-for-service	Fee-for-service	Fee-for-service

Data resource: 2013 China Health Statistics Yearbook, Websites of Ministry of Human Recourse and Social Security and National Heath and Family Planning Commission of China

Benefits and financing vary across cities/counties and medical institute levels

In general, existing literature investigating the impacts of health insurance on health-care utilization in China supports the conclusion that insurance increases the utilization of health services (Gao et al. 2007; Wagstaff and Lindelow 2008). Using data from the China Health and Nutrition Survey (CHNS), a 2009 study examined the impact of the NCMS on the utilization of health services. The authors found that participating in the NCMS improved the use of preventive care, but did not increase the utilization of formal medical services (Lei and Lin 2009). According to a recent study, the hospitalization costs of urban patients with mental illnesses in China (5731.44 RMB, equivalent to 19.5% of the average urban disposable income of 29,381 RMB) was significantly higher than that of rural patients (3687.54 RMB, equivalent to 37.3% of the average rural disposable income of 9892 RMB) (Feng et al. 2014; NHFPC 2015). However, the empirical literature on the utilization of mental health services in China is still quite limited, and does not address disparities. Most existing literature uses survey data focused on one health insurance scheme, or exclusively on rural or urban residents. As discussed earlier, differences in insurance schemes only roughly capture the differences in insurance coverage across China.

In this study, we used 10 years (2005–2014) of hospital electronic health records (EHRs) to examine the effects of the three main health insurance schemes on the utilization of mental health services in China. EHR data are not subject to recall bias; the actual RR was constructed to measure health insurance coverage, and is a more accurate measure than the type of health insurance.

## Method

### Study design and study sample

We designed a retrospective cohort study of patients hospitalized with mental illnesses to assess rural–urban disparities in the utilization of mental health services, and the role of health insurance. Our study site included two major psychiatric hospitals in Shandong, China: the Shandong Center for Mental Health (SCMH) and the Daizhuang Psychiatric Hospital (DPH).

Shandong is the second-most populous province in China. With more than 97 million people, its population accounts for 7.2% of the national total. In the province, 44 million people (44.99% of the population) live in rural areas, which is consistent with the national rural and urban population distribution (China Health Statistics Yearbook). In total, there are 1158 general hospitals providing mental health services and 53 psychiatric hospitals in the province. SCMH is the only provincial psychiatric hospital, and DPH is one of the oldest psychiatric hospitals in the province. These two psychiatric hospitals serve almost 10% of all mental health patients every year in Shandong (HFPCSD 2015), and accept patients from all over the country. In addition, health insurance policy regulations in Shandong Province are consistent with national health insurance policy regulations.

Our study population was identified using patients’ primary diagnosis, as recorded in the two hospitals’ EHRs, which routinely record information on patients’ socio-demographic characteristics (e.g., gender, age, marital status, and place of residence); clinical characteristics (diagnosis based on the International Classification of Diseases, 10th version, ICD-10); cost-related information (e.g., the cost of drugs, examinations, the hospital bed, and total hospital costs); and insurance information (types of insurance, costs reimbursed by health insurance, and OOP costs when discharged). A major strength of the EHR data is that they document all inpatient expenses incurred during hospitalization in a detailed, itemized, and reliable way. All data are collected and recorded by hospital registries, physician workstations, and the insurance settlement departments, with minimal recall bias. Descriptive statistics of variables are presented in “Appendix 1”.

**Fig. 2:**
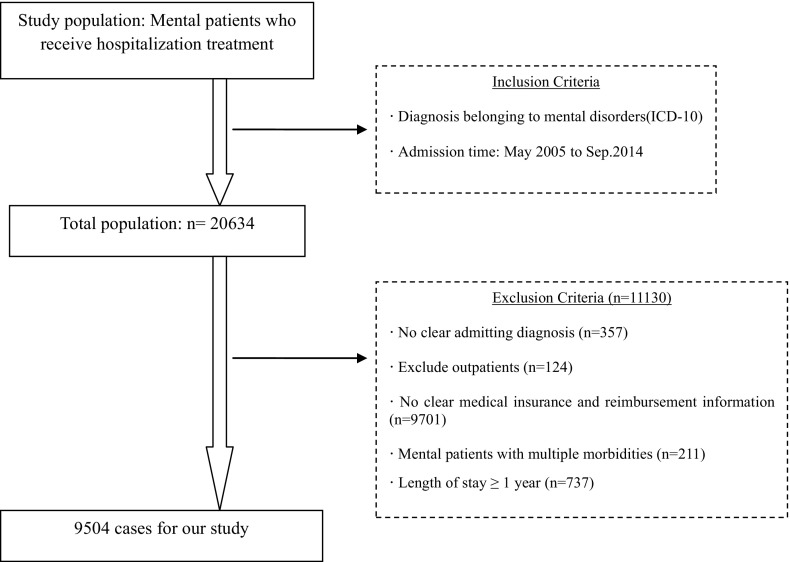
Flow chart of inclusion and exclusion criteria of published studies

Figure 2 outlines our sample selection process. Mental health patients were included in our samples using two criteria. First, mental illness was the primary diagnosis (ICD-10 codes: F0, organic mental disorders including symptomatic disorders and dementia; F1, mental and behavioral disorders due to the use of psychoactive substances; F2, schizophrenia, schizotypal, and delusional disorders; F3, mood [affective] disorders; F4, neurotic, stress-related, and somatoform disorders; F5, behavioral syndromes associated with physiological disturbances and physical factors; F6, disorders of adult personality and behavior; F7, mental retardation; F8–9, others). Second, the patients were discharged from these two hospitals between May 2005 and March 2014. Inpatients without a clear initial diagnosis or clear medical insurance and reimbursement information were excluded. We also excluded cases in which the length of stay (LOS) exceeded 1 year. This resulted in a sample of 9504 inpatient cases.

### Reimbursement ratio variable

As discussed earlier, there are large variations in insurance coverage for mental health services, not only across different insurance programs but also within the same insurance program. Therefore, in addition to insurance type, we used actual RR in this study to measure health insurance. Compared with the conventional measures such as insurance type or policy, RR is a more accurate measure in determining how generous the health insurance is in terms of coverage (Li and Zhang 2013; Goldman et al. 2006).

RR was defined as the percentage of costs reimbursed by health insurance over total medical costs (TC). The exact equation being: RR = (TC-OOP)/TC * 100%. TC represents total medical costs, and OOP represents out-of-pocket payments made by individuals for their health-care services. In our sample, the actual RR ranged from 0 to 100%.

### Statistical analyses

All statistical analyses were based on a pooled dataset with information from the two EHR databases on the selected sample. Descriptive analyses, the Mann–Whitney U test, and multiple regressions combined with the Poisson model were employed to examine the effect of health insurance on the utilization of mental health services.

Descriptive analyses were used to describe patient characteristics and use of mental health services, measured by LOS, frequency of hospitalization, and hospitalization costs. We chose to analyze the utilization of mental health inpatient services using these three measures as they are the most commonly used for inpatient resource use (Feng et al. 2014; Garland et al. 2005; Xu et al. 2014). While there is no single perfect way to measure inpatient services, all three measures were used to better capture the potential impacts of health insurance on inpatient service utilization. Differences in the utilization of mental health services by urban and rural inpatient status were examined using the Mann–Whitney U test. Because many mental disorders involve frequent relapse, there might be multiple treatment episodes for the same patient every year. Therefore, we also estimated the frequency of hospitalization per patient per year.

We used the following empirical specifications to examine disparities in the utilization of mental health services:

1. $$ {\text{Utilization}} =\upalpha +\upbeta_{1} *X +\upbeta_{2} *Insurance +\upbeta_{3} *RR +\upvarepsilon $$
2. $$ Utilization =\upalpha +\upbeta_{1} *X +\upbeta_{2} *Insurance +\upbeta_{3} *RR +\upbeta_{4} *\left( {RR*Insurance} \right) +\upvarepsilon $$

where X includes type of hospital, gender, age, marital status, and diagnosis (schizophrenia, depression, and others, defined based on ICD-10); area of residence (urban or rural, defined based on the place of household registration); and admission severity (stable, severe, and critical, defined based on ICD-10). Mental health utilization is measured by hospitalization cost, LOS, and frequency of hospitalization. The insurance variable is denoted by three dummy variables: UEBMI, URBMI, and NCMS. In addition, we also measure health insurance using the reimbursement ratio (RR). The interaction terms (RR * Insurance) in Model (2) refers to RR * UEBMI, RR * URBMI, and RR * NCMS. (See “Appendix 2” for variable definitions.)

The LOS and hospitalization costs showed a seriously skewed distribution. Therefore, log transformation of these dependent variables (e.g., LOS and hospitalization costs) were used in the estimations. Moreover, as a count variable, the distribution of the frequency of utilization was strongly skewed to the right. The Poisson model was used to analyze the effect of health insurance on the frequency of utilization; 18.0 SPSS software was used for the statistical analyses with an alpha level of 0.05.

## Results

The descriptive statistics of our sample are presented in Table 2. Of the patients in our sample, 52.4% (4980) were from rural areas and 47.6% (4524) from urban areas; 46.7% (4443) were male; 70.3% (6678) were younger than 59 years; and 28.5% (2712) were diagnosed with schizophrenia. Only 33.8% (3215) had any form of insurance, partly because of the relatively recent implementation of the URBMI in 2007 and the NCMS in 2003. Also, it is likely that many patients in our sample were seeking mental health care outside of their cities/towns of residence, and therefore their treatment was not covered by insurance.

**Table 2:** Sample descriptive statistics (N = 9504)

Items	Number	Percentage (%)
Type of hospital		
Municipal	3515	37.0
Provincial	5989	63.0
Gender		
Man	4443	46.7
Woman	5061	53.3
Age		
≤ 45	3611	38.0
45–59	3067	32.3
≥ 60	2826	29.7
Marital status		
Have a spouse	3174	33.4
No spouse	6330	66.6
Occupations		
No	282	3.0
Farmer	4002	42.1
Employee	1941	20.4
Retired	724	7.6
Students	881	9.3
Others	1674	17.6
Residence		
Urban	4524	47.6
Rural	4980	52.4
Diagnosis		
Schizophrenia	2712	28.5
Depression	1213	12.8
Other	5579	58.7
Insurance^a^		
No	6289	66.2
Yes	3215	33.8
Admission severity		
Stable	4278	45.0
Severe	112	1.2
Critical	18	0.2
Missing	5096	53.6

^a^The percentage of patients receiving reimbursements from health insurance was low, because the implementation of URBMI was late (2007) and/or there was a time-lag with NCMS policy and/or many patients sought mental health care outside their city of residence (and insurance coverage was not portable for them)

Table 3 describes the utilization of mental health services among insured and uninsured patients, and in urban and rural areas. Utilization rates, measured by inpatient hospital costs, OOP payments, the RR, LOS, and frequency of hospitalization, were found to be significantly higher among urban than rural patients, both among insured and uninsured groups. Consistent with this finding, as shown in Table 4, the utilization rate of patients covered by the NCMS (i.e., rural patients) was significantly lower than for patients under the URBMI and the UEBMI (i.e., urban patients).

**Table 3:** Mental health utilization among insured and uninsured patients

Items	Number	Hospitalization cost (RMB)	Out of pocket payment (RMB)	Reimbursement ratio (%)	LOS (d)	Frequency of hospitalization
Insured	3215	11,252.89	3980.69	56.88	70	2
Rural	1179	6175.62	3060.69	48.17	25	2
Urban	2036	16,900.59	5004.06	69.18	121	3
*P* value		< 0.01	< 0.01	< 0.01	< 0.01	< 0.01
Urban–rural disparity	–	10,724.97	1943.37	21.01	96	1
Uninsured	6289	9509.40	9509.40	0	54	1
Rural	3801	8997.74	8997.74	0	46	1
Urban	2488	9893.27	9893.27	0	60	1
*P* value		< 0.01	< 0.01	< 0.01	< 0.01	0.85
Urban–rural disparity	–	895.53	895.53	0	14	0

^a^The data in the table displays the mean value

**Table 4:** Mental health utilization by insurance type

Items	Hospitalization cost (RMB)	Out of pocket cost (RMB)	Reimbursement ratio (%)	Frequency of hospitalization	LOS (d)
UEBMI	19,054.25	5365.41	74.64	3.96	137.52
URBMI	9497.78	3762.01	59.23	2.27	63.70
NCMS	6175.62	3060.69	48.04	1.91	24.99
*P* value	0.033	0.000	0.000	0.002	0.000

^a^The data in the table displays the mean value

**Fig. 3:**
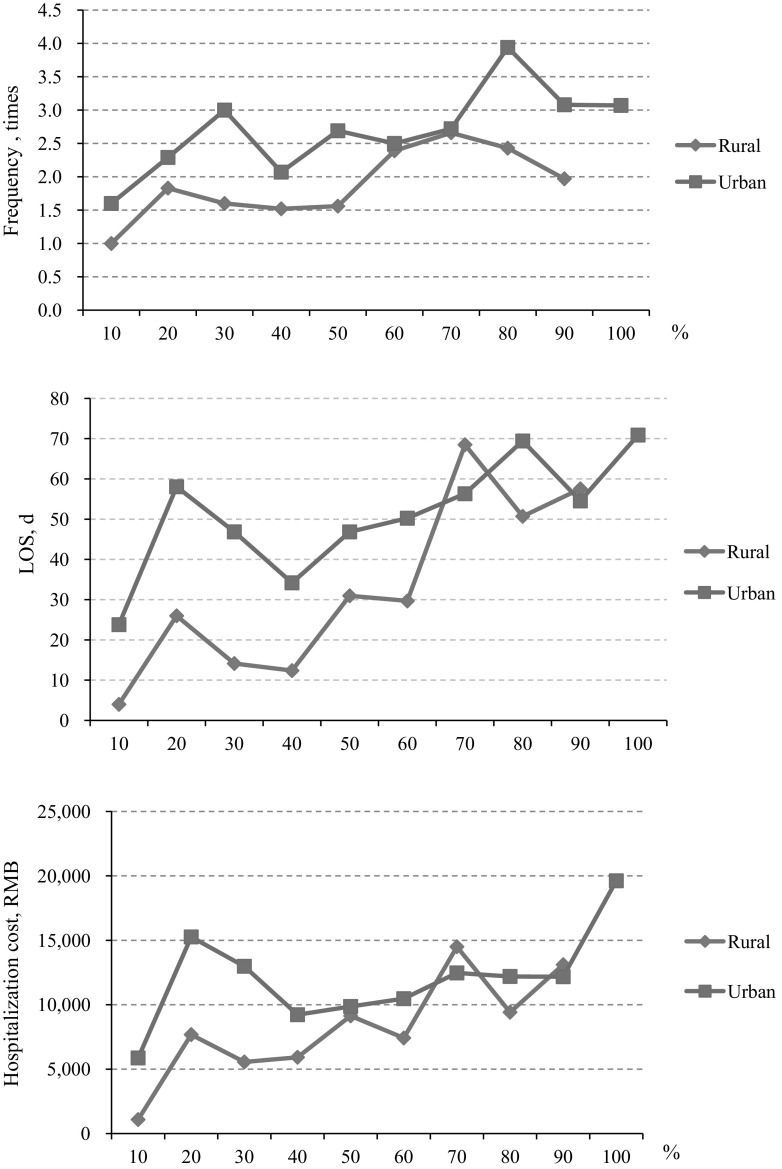
Mental health utilization by reimbursement ratio

Figure 3 illustrates the trends of mental health patients’ hospitalization costs by RR. As shown in the figure, the hospitalization costs, LOS, and the frequency of hospitalization of urban patients was found to be consistently higher than that of rural patients at almost all RR levels. However, when the RR increases, urban–rural differences in the utilization of mental health services significantly decreases. Moreover, for both rural and urban patients, hospitalization costs per case and LOS increased with the RR.

The multiple regression results for the use of mental health services, based on Models (1) and (2), are presented in Table 5. Urban patients tended to utilize more mental health services than did rural patients. This utilization was also found to be significantly associated with the RR: when the RR increases, the utilization of mental health inpatient services also increases. In addition, analysis of the RR’s interaction with different types of health insurance indicated that an increase in RR was significantly associated with an increase in rural patients’ utilization of mental health services. Moreover, a univariate analysis showed that LOS was significantly associated with higher inpatient costs.

**Table 5:** Multiple regression results for mental health utilization

Variables	Frequency of hospitalization		LOS		Hospitalization cost	
	Model 1	Model 2	Model 1	Model 2	Model 1	Model 2
Type of hospital	0.183	0.160	0.196	0.177	0.026	0.019
Gender	0.031	0.044	0.157	0.161	0.042	0.044
Age	0.069	0.070	0.111	0.114	0.026	0.026
Marital status	− 0.219^a^	− 0.220^a^	− 0.599^a^	− 0.609^a^	− 0.177^a^	− 0.179^a^
Diagnosis	− 0.007	− 0.009	0.001	0.000	− 0.007	− 0.007
Residence	− 0.591	− 0.647	− 0.114	− 0.168	− 0.053	− 0.036
Admission severity	0.256	0.266	0.665	0.674	0.263^a^	0.266^a^
Types of insurance	0.179^a^	0.076	0.123	0.116^a^	0.061	0.016^a^
URBMI in 2007	0.814	− 1.690	0.132	0.501	0.058	− 0.091
RR	0.095^a^	0.090	0.102^a^	0.094^a^	0.066^a^	0.064^a^
RR * NCMS	–	0.329^a^	–	0.121^a^	–	0.011^a^
RR * URBMI	–	0.036	–	0.033	–	0.003
RR * UEBMI (Control group)	–	–	–	–	–	–
R^2^	0.159	0.166	0.106	0.107	0.228	0.229

The coefficients could not be read in the same way because different regression models were used for each dependent variable

^a^*P* value < 0.05

## Discussion

In China, more than 173 million people suffer from mental disorders (Phillips et al. 2009). Partly due to the high economic burden of treatment, nearly 90% of patients with mental disorders, especially those in rural areas, have never sought any professional treatment (Phillips et al. 2009). In poor or remote areas, patients with mental disorders might even fall victim to superstitious activities (Wang 2014).

Our study provides clear empirical evidence of rural–urban disparities in the utilization of mental health services in China: mental health patients in rural areas, both insured and uninsured, use significantly less inpatient mental health care than do those in urban areas. One possible explanation for this is that compared with urban residents, rural residents use more outpatient, and less inpatient, services, particularly among uninsured populations (Liu et al. 2007). The relatively low cost of outpatient visits in rural areas may encourage people to seek out physicians for consultation but avoid inpatient services. Moreover, the limited availability of specialists in rural areas, as well as the greater travel time and distance involved in seeking care, may contribute to this difference. On the other hand, outpatient services may be a way to triage rural patients with mental health services and keep patients from flocking to cities for medical care, which could relieve the stress faced by the mental health system in China.

Another possible explanation is that rural residents are not able to afford high inpatient costs, due to the low insurance reimbursement level of rural patients. Under the current insurance system, the average RR of rural health insurance is more than 20% lower than that of insurance provided in urban areas; rural residents face much higher OOP expenses. For rural residents, even though the RR of NCMS for inpatient treatment has been increasing since 2003, the ratio is still low, at around 50% (Meng et al. 2012). This represents a significant economic burden for rural patients. Existing literature shows that NCMS did not reduce the economic burden of illnesses for patients; health-care utilization among rural residents remains primarily determined by socioeconomic factors (Wagstaff et al. 2009; Li and Zhang 2013; Yu et al. 2010; Shi et al. 2010). The results of our study are consistent with these findings. Another recent study, focusing on general health-care utilization, also shows that compared with insured urban respondents, insured rural respondents are less likely to be hospitalized, although health service utilization substantially improves alongside an increase in the reimbursement level (Fu et al. 2014).

Income disparities are cause for concern in China: per capita disposable income in 2014 was $4738.9 for urban residents but only $1595.5 for rural residents (NHFPC 2015). If no adequate measures are taken to develop a uniform standard health insurance system for urban and rural residents, the growing rural–urban income disparity may exacerbate disparities in health service utilization, including for mental health (Fu et al. 2014).

Health insurance in China is not portable. In China, the pooling district of the basic medical insurance scheme is based at the county or city level. Therefore, if a rural mental health patient seeks care in a hospital outside the pooling district, he or she will face extremely complex reimbursement procedures and may simply not get reimbursed. Meanwhile, China’s health-care budget is heavily skewed toward physical diseases and urban areas (WHO 2010). Most of the large psychiatric hospitals, too, are located in urban areas. In our study sample, about two-thirds of mental health inpatients were not eligible to use public health insurance to pay for their hospital treatment. The nonportability of health insurance is a major barrier to rural patients seeking mental health care.

The expansion of public health insurance has been an important item on the health-care reform agenda in China. To date, the country’s public health insurance covers more than 90% of its citizens. But reform should also address issues such as the wide variation in coverage across different types of insurance, and particularly between urban and rural areas. Our study provides insights on the role of health insurance and RRs in addressing the issue of rural–urban disparities in mental health care.

In our sample, regardless of rural or urban residence status, insured inpatients tend to use more mental health services than uninsured patients; higher RRs are associated with greater utilization of mental health services. This result is consistent with earlier studies (Zhou et al. 2009). An increase in utilization may be explained by the moral hazard effect. Indeed, health economics literature has pointed out that the moral hazard effect is larger for mental health services than for general health services (Frank et al. 2001). However, for mental health patients in rural China, the moral hazard is likely outweighed by unmet needs. A large proportion of mental health patients in rural China are poor and cannot afford inpatient treatment (Xu et al. 2016). People often cite financial barriers, such as the cost of care or lack of health insurance coverage, as reasons for not receiving health care, including for mental health (Sareen et al. 2007; Mojtabai 2005; Garland et al. 2005; Fortney et al. 2003).

Consistent with the existing literature, our study suggests that an increase in the RR level of the public health insurance scheme could lead to substantial improvements in the utilization of mental health inpatient services for both rural and urban patients (Weathers and Stegman 2012; Simon et al. 1996; Stein et al. 2000; Lo Sasso et al. 2006). More importantly, such an increase would be larger among rural patients. This result indicates that increasing the RR of NCMS would also lead to a reduction in rural–urban disparities in mental health utilization.

However, a small-scale increase in the RR of the NCMS is less likely to result in an improvement in the utilization of rural mental health services. As discussed earlier, the current co-payment rate under UEBMI is about 80%, significantly higher than NCMS (about 30%). In order to reduce rural–urban disparities in health-care utilization, the Chinese government launched the gradual merger of the URBMI and NCMS systems as of 2016, in order to establish a uniform basic medical insurance system for rural and urban residents. During this process, policy makers in China would need to consider significantly improving the insurance coverage (particularly in rural areas), including increasing the RR and the annual reimbursement limit, and expanding coverage services to mental inpatients to cover the unmet need (Wang et al. 2007a, b). Severe mental illnesses have now been classified as, simply, severe illnesses, which implies that patients with a severe mental disease can apply for a higher reimbursement if they receive treatment in the local, designated hospital. However, as we discussed earlier, limited specialists and the limited portability of health insurance pose serious obstacles to rural patients. In addition, our results show that when the RR increases, both the frequency of hospitalization and LOS decrease. This may imply that patients with a higher RR achieved a better treatment outcome at discharge and/or received more adequate treatment. Our result provides support for the current policy piloted in some cities (i.e., the city of Zhongshan in Guangdong Province), in which the RR is set higher during a given period, e.g., the initial 36 months (ZHRSSB 2015; Lu et al. 2004).

Our study is subject to limitations. First, we used inpatient administrative data to examine rural–urban disparities in the utilization of mental health services. Our data do not provide information on outpatient and community mental health services, which are quite important in China, particularly considering China’s widespread “free medication” program (“686” program) that provides a considerable amount of mental health services to people with severe mental disorders at the community level. Future studies should look at both outpatient and community mental health services. Second, patients with private health insurance were excluded from our study, given that private insurance in China is still nascent, mainly targeting high-income households and covering only about 7% of the population (Wang et al. 2013). Third, we used data from two large mental health hospitals in Shandong Province, and the sample may not be representative of the entire country. However, the economic levels of the areas where these two hospitals are located reflect the national average. In these areas, average per capita disposable income in 2014 was 20,864 RMB (34.3% of GDP per capita) versus the national 20,167 RMB (42.7% of GDP per capita); per capita health expenditure was 1711 RMB (3.3% of per capita income) versus the national 1807 RMB (3.2% of per capita income), and the per capita hospital cost was 6698 RMB (13.0% of per capita income) versus the national cost of 6980 RMB (12.4% of per capita income) (China Health Statistics Yearbook).

## Conclusions

Large rural–urban disparities in the utilization of mental health services exist in China, with financial concerns being a major barrier to rural patients seeking mental health care. Health insurance coverage, particularly the reimbursement ratio, could be a powerful policy tool to influence people’s health-care utilization. In order to improve access to and reduce rural–urban disparities in mental health care, future health-care reform in China should consider expanding mental health coverage, particularly in rural areas, as well as making health insurance portable.

## Appendix 1

**Table:** Descriptive statistics of variables

Variables	Mean	Standard deviation	Minimum	Maximum
Age (year)	46.23	15.98	18	105
RR (%)	46.50	35.43	2	100
Hospitalization cost (RMB)	10,511.85	2544.58	18.1	585,394.47
Out of pocket payment (RMB)	4163.18	994.40	0	368,335.18
LOS (d)	66.27	235.43	1	107
Frequency of hospitalization	2.24	3.733	1	12

## Appendix 2

**Table:** Variable definitions

Items	Assignment
Type of hospital	
Municipal	1
Provincial	2
Gender	
Man	1
Woman	2
Age	Actual values
Marital status	
Have a spouse	1
No spouse	2
Residence	
Urban	1
Rural	2
Diagnosis	
Schizophrenia	1
Depression	2
Other	3
Admission severity	
Stable	1
Severe	2
Critical	3
Types of insurance	
NCMS	1
URBMI	2
UEBMI	3
RR	Actual values
